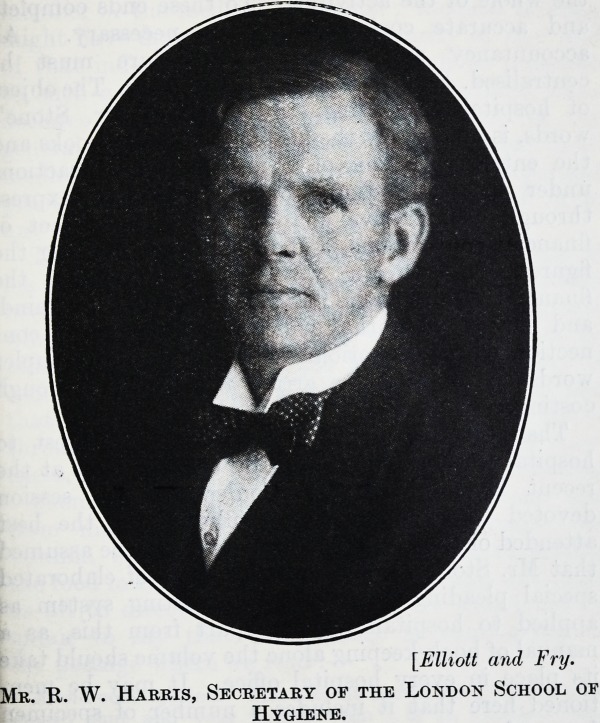# London School of Hygiene and Tropical Medicine

**Published:** 1924-08

**Authors:** 


### LONDON SCHOOL OF HYGIENE AND TROPICAL MEDICINE.

Mr. R. W. Harris, who retired from the office of Assistant Secretary in the Ministry of Health in February, 1922, has been appointed Secretary of the

[*Elliott and Fry.*

MR. R. W. HARRIS, SECRETARY OF THE LONDON SCHOOL OF HYGIENE.

London School of Hygiene and Tropical Medicine. The Director of the School is Dr. Andrew Balfour, C B., C.M.G., and an article on the scope of the work of the School, which is not yet built, appeared in our last issue. Mr. Harris has been for the past two years a regular contributor to the HOSPITAL AND HEALTH REVIEW.

## Figures and Tables

**Figure f1:**